# Intermediate and high peri-operative cardiac enzyme release following isolated coronary artery bypass surgery are independently associated with higher one-year mortality

**DOI:** 10.1186/1749-8090-1-20

**Published:** 2006-08-15

**Authors:** N Newall, AY Oo, ND Palmer, AD Grayson, TJ Hine, RH Stables, BM Fabri, DR Ramsdale

**Affiliations:** 1 Department of Cardiology, Arrowe Park Hospital, Wirral, UK; 2 Department of Cardiothoracic Surgery, The Cardiothoracic Centre, Liverpool, UK; 3 Department of Cardiology, The Cardiothoracic Centre, Liverpool, UK; 4 Department of Clinical Governance, The Cardiothoracic Centre, Liverpool, UK; 5 Department of Clinical Biochemistry, The Royal Liverpool and Broad Green University Hospital, UK

## Abstract

**Background:**

The relationship between cardiac enzyme (CE) release following coronary artery bypass surgery (CABG) and medium term outcome is unclear. We sought to determine the relationship between post-operative CE release and one-year survival following isolated CABG.

**Methods:**

Over three years 3,024 consecutive patients underwent isolated CABG. Patient characteristics were prospectively recorded in a cardiac surgical database. CE release, taken as the highest single measurement recorded in the first 24 hours post-op, was abstracted from an electronic archive. All cause mortality was taken from a national registry of deaths.

**Results:**

Data were complete for 2,860 (94.6%) patients. CK-MB isoenzyme (reference range 5–24 U/l) was recorded in 2,568 (89.8%), total CK in 292 (10.2%).

CE release three or more times the upper limit of the reference range (ULR) were recorded in 498 (17.4%) patients, 163 (5.7%) patients had CE more than six times ULR. There were 122 deaths (4.3%). Cox proportional hazards analysis showed that CE release 3–6 times ULR (adjusted HR 2.1 [95% CI: 1.6 to 2.6], p = 0.002) and CE release six or more times the ULR (adjusted HR 5.0 [95% CI: 4.5 to 5.4], p < 0.001) were independently associated with increased one-year mortality.

**Conclusion:**

Cardiac enzyme release following CABG is associated with increased one-year all-cause mortality. The definition of peri-operative myocardial infarction following CABG should include elevation of CK-MB three or more times the upper limit of normal.

## Background

Myocardial necrosis causing release of cardiac enzymes (CE) following otherwise uncomplicated percutaneous coronary intervention has been associated with increased medium term mortality [1,2]. Myocardial necrosis occurs frequently in patients undergoing cardiac surgery. Putative mechanisms of cardiac myonecrosis include diffuse ischaemic injury during cardiopulmonary bypass, reperfusion injury, systemic inflammatory activation [3], embolisation of atheromatous material during coronary manipulation [4], and early graft occlusion [5], as well as atrial cannulation and left ventricular venting [6].

Defining peri-operative myocardial infarction following cardiac surgery has proven problematic due to the difficulty in interpreting pain, electrocardiographic (ECG), and haemodynamic changes in the early post operative phase. Peri-operative ECG changes such as T wave inversion, left or right bundle branch block, and new Q waves have poor sensitivity and specificity for myocardial infarction [7-9], and appear not to be associated with reduced medium and long term survival [9,10]. Cardiac specific biochemical markers of myocardial injury are attractive surrogates for clinical outcomes following cardiac surgery, but lack robust thresholds for association with a diagnosis of myocardial infarction and reduced survival [11]. The most recent European Society of Cardiology/American College of Cardiology joint guideline did not specify threshold values of post-operative cardiac enzyme release that defined myocardial infarction following coronary artery bypass graft (CABG) surgery [12].

The current consensus among cardiac surgeons and cardiologists is that post CABG CK-MB elevation of least 5 times the upper limit of reference range (ULR) marks the threshold of prognostic significance [13]. However considerable variation exists between the threshold values of post CABG CK-MB release and subsequent mortality observed among patients recruited in randomised controlled trials [14,15], and unselected registries [8,16,17]. The clinical implications of recently revised diagnostic criteria for myocardial infarction have been recognised and the need for prognostic evaluation of peri-operative CE release specifically emphasised [18,19].

We sought to determine the association between CE release and survival at one-year, and to identify pre-operative predictor variables associated with increased CE release following isolated CABG.

## Methods

### Patient population and data

An observational cohort study was performed. Using a prospectively recorded cardiac surgical database we identified 3,024 consecutive patients, undergoing isolated CABG between 1^st ^January 1999 and 31^st ^December 2001 at the Cardiothoracic Centre-Liverpool. Patients undergoing surgery that involved heart valve repair or replacement, resection of a ventricular aneurysm or other surgical procedure were not included. The study was approved by the local ethical committee.

All data were collected prospectively during the patient admission as part of routine clinical practice (see Table 1). Data collection methods and definitions have been described in detail previously [20].

**Table 1:** Patient characteristics

	Study Population (n = 3,024)
Age at operation (years)	64.3 (58.4 to 70.0)
Female sex (%)	19.9
Body mass index (kg/m^2^)	27.6 (25.3 to 30.4)
Angina class IV (%)	32.9
Current smoker (%)	15.8
Previous myocardial infarction (%)	44.2
Diabetes (%)	17.6
Hypercholesteroleamia (%)	83.8
Hypertension (%)	55.3
Peripheral vascular disease (%)	13.8
Cerebrovascular disease (%)	8.2
Renal dysfunction (%)	2.7
Respiratory disease (%)	31.6
Ejection fraction <30% (%)	8.7
Three-vessel disease (%)	81.3
Left main stenosis >50% (%)	20.1
Prior cardiac intervention (%)	5.1
Prior cardiac surgery (%)	2.5
Urgent procedure (%)	16.9
Emergent procedure (%)	1.4

Continuous variables are shown as median with 25^th ^and 75^th ^percentiles.

Categorical variables are shown as a percentage.

### Cardiac enzyme data

During the study period it was our practice to routinely request cardiac enzymes on admission to the intensive care unit and on the morning of the first post operative day. All assays were performed at 37°C. The assays used were CK-MB immuno assay (Roche, reference range 5–24 U/l at 37°C) and total CK (Roche, reference range <190 U/l in men, <167 U/l in women at 37°C). CE results for the first 24 hours post-op were taken from a routinely recorded electronic clinical biochemistry archive, blind to survival and other clinical data. When more than one CE result existed the highest value of CK-MB isoenzyme was selected in preference to the highest value of total CK.

### One-year mortality data

Patient records were linked to the National Strategic Tracing Service (NSTS), which records all-cause mortality in the United Kingdom. To establish current vital status, at one-year of follow-up, patients were matched to the NSTS based on patient name, National Health Service unique number, date of birth, gender, and postcode.

### Statistics

Continuous variables were not normally distributed and are shown as median with 25th and 75th percentiles. Categorical data are shown as percentages. Deaths occurring as a function of time, at one-year of follow-up were described using the product limit methodology of Kaplan and Meier [21]. Cox proportional hazards analysis was used to identify independent risk factors for increased one-year mortality and to calculate risk adjusted Kaplan-Meier survival curves [22]. Multivariate logistic regression was used to identify independent preoperative risk factors for CE release above the threshold level associated with increased one-year mortality. The C statistic (equivalent to the area under the receiver operating characteristic curve) and Hosmer-Lemeshow goodness-of-fit statistic were calculated to assess the performance and calibration of the logistic model respectively [23]. All variables listed in Table 1 were included as potential risk factors in both the Cox proportional hazards and logistic regression analyses. In all cases a p value < 0.05 was considered significant. All statistical analysis was performed with SAS for Windows Version 8.2.

## Results

Patient characteristic data were complete for all 3,024 patients (Table 1). Follow up for survival up to and including 31st December 2002 was complete. Cardiac enzymes were recorded in 2,860 (94.6%) who were included in the analysis. Patients without CE results (n = 164) did not have statistically significant differences in baseline characteristics compared to patients with CE results, and had 3% one-year mortality.

Among the 2,860 patients with CE results, 2,568 (89.8%) had CK-MB isoenzyme and 292 (10.2%) total CK only performed within the first 24 hours post-op. The median CK-MB iso-enzyme result was 36 U/l (25^th ^and 75^th ^percentiles: 24 to 60 U/l). The median total CK was 248 U/l (25^th ^and 75^th ^percentiles: 213 to 285) for men, and 247 U/l (25^th ^and 75^th ^percentiles: 199–283) for women. Four hundred and ninety eight (17.4%) patients had cardiac enzymes three or more times the ULR. Two hundred and twenty nine (8.0%) patients had CE five or more times the ULR. One hundred and sixty three (5.7%) patients had CE more than six times the ULR, while 59 (2.1%) had cardiac enzymes ten or more times the ULR.

There were 122 (4.3%) deaths during the first post-operative year among the 2,860 patients with CE results. Cox proportional hazards analysis revealed that CE release >6 times the ULR (adjusted hazard ratio (HR) 5.0 [95% CI: 4.5 to 5.4], p < 0.001) and CE release 3 to 6 times the ULR (adjusted HR 2.1 [95% CI: 1.6 to 2.6], p = 0.002) were independent predictors of increased mortality at one-year. The crude mortality was identical for cardiac enzymes 3 to 5 times, and 5 to 6 times the ULR at 4.8%. When CE release 3 to 5 times the ULR was offered to the model the magnitude of association with increased one-year mortality was not substantially changed (adjusted HR 1.9, p = 0.015) confirming that the association for the group of patients with CE release 3 to 6 times the ULR is not solely driven by the highest values of CK-MB in this band.

Other independent risk factors for one-year mortality included poor ejection fraction (< 30%), increasing age, pre-operative renal dysfunction, peripheral vascular disease, prior cardiac surgery, emergency surgery, and female sex (Table 2). Other variables listed in Table 1 were not found to be associated with increased mortality.

**Table 2:** Independent risk factors for increased one-year mortality

	Hazard Ratio	95% Confidence Intervals	p Value
CE release >6 times the ULR	5.0	4.5 to 5.4	<0.001
Ejection fraction <30%	3.1	2.7 to 3.5	<0.001
Age at operation *	1.06	1.03 to 1.08	<0.001
Renal dysfunction	2.6	2.0 to 3.2	<0.001
Peripheral vascular disease	2.1	1.7 to 2.5	0.002
CE release 3 to 6 times the ULR	2.1	1.6 to 2.6	0.002
Prior cardiac surgery	2.9	2.2 to 3.7	0.006
Emergent procedure	2.8	2.0 to 3.6	0.007
Female sex	1.5	1.1 to 1.9	0.041

CE, Cardiac enzyme; ULR, Upper limit of reference range.

* per unit increase.

**Figure 1:**
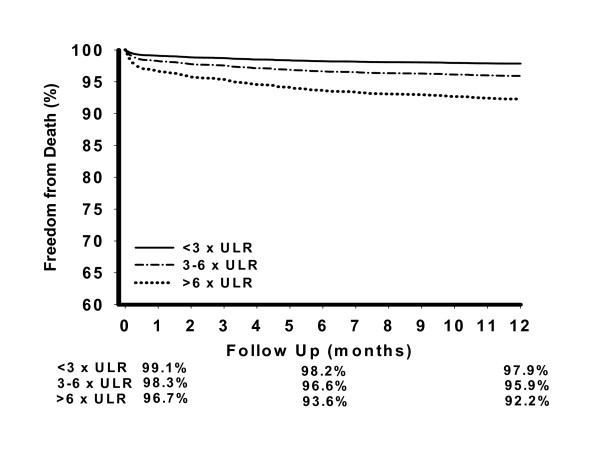
Adjusted one-year survival following coronary artery bypass surgery for cardiac enzyme release <3 times, 3 to 6 times, and >6 times the ULR. Data adjusted for risk factors listed in table 2.

The relationship between CE release and death within the first post-operative year are displayed in the risk adjusted survival curves (Figure 1). The independent association between increased mortality and CE release 3 to 6 times the ULR and CE release >6 times the ULR was unaffected by restricting analysis to patients with CK-MB isoenzyme results, with adjusted HR 2.3 (p = 0.001) and adjusted HR 6.2 (p < 0.001) respectively.

All the patient characteristics listed in Table 1 were offered to a multivariate logistic regression analysis. We found that female sex, extent of coronary disease, peripheral vascular disease, hypercholesterolaemia, and hypertension were independent predictors of CE release three or more times the ULR (Table 3).

**Table 3:** Independent risk factors for cardiac enzyme release >= 3 times the ULR

	Odds Ratio	95% Confidence Intervals	p Value
Female sex	1.72	1.37 to 2.15	<0.001
Triple-vessel disease	1.48	1.13 to 1.94	0.005
Peripheral vascular disease	1.39	1.07 to 1.81	0.013
Hypercholesterolaemia	1.39	1.04 to 1.86	0.024
Hypertension	1.24	1.02 to 1.52	0.034

ULR, Upper limit of reference range.

C 0.61, Lemeshow-Hosmer goodness of fit P = 0.76

## Discussion

This study shows a highly statistically significant, and clinically meaningful independent association between CK-MB release within the first 24 hours following isolated CABG surgery and increased mortality within the first post-operative year. Although the highest risk of death is associated with greater CE release we have demonstrated that the independent association between CE release and death is not confined to the few individuals with highest values of CE release. To the best of our knowledge this is the first study to show that modest levels of post-CABG CE release 3 to 6 times the ULR are independently associated with higher one-year mortality.

CE release three or more times the ULR occurred in approximately one in six of the patients in this study. Such patients were more frequently female, had more target vessels, and peripheral vascular disease. These associations may be due to more challenging surgical anatomy [24], and more widespread and advanced atherosclerotic disease respectively. However the low c statistic (0.61) for the multivariate pre-operative characteristic model, suggests that a significant proportion of the risk of CE release three or more times the ULR may be accounted for by other variables not included in the present analysis.

We chose to use a CK-MB immuno assay as this had been historical practice at the Cardiothoracic Centre-Liverpool. Although CK-MB estimation remains the most widely used post revascularisation assay our results may have been strengthened had we used more sensitive and specific enzymes assays such as troponin T or I [25,26]. However recent comparisons between CK-MB and tropoinin I in predicting irreversible myocardial injury, detected by cardiac magnetic resonance imaging, following CABG did not suggest that peak troponin I was superior to peak CKMB [27]. Although troponin assays are more closely associated with early graft occlusion [28,29], further investigation is required to confirm their independent prognostic superiority for survival following surgical re-vascularisation procedures.

Earlier studies suggested that the association between increased medium term mortality and peri-operative CE release following CABG surgery was confined to those individuals with CE release >5 times the ULR [9,14,15], or CE release >10 times the ULR [16], and that an independent association between CE release 3 to 5 times the ULR and mortality was only apparent after long term follow up [17]. Our data shows that although CE release >6 times the ULR is more strongly associated with death in the first post-operative year, lower levels of CE release are also associated with increased one-year mortality. The discrepancy between the findings in this and earlier studies may in part be explained by a more inclusive and completely described cohort, and lower heterogeneity in the timing and type of cardiac enzyme assay used in our study.

Both Klatte [14] and Costa [15] found CK-MB release 5 times or more the ULR was associated with an increased risk of mortality at 6-months and one-year respectively. Both studies however, originated from randomised control trials and excluded high-risk patients such as left main stem stenosis, impaired left ventricular function, cerebrovascular disease, and significant renal impairment. Our own study population, however, included 20.1% with left main stem stenosis, 8.7% with ejection fraction <30%, 8.2% with cerebrovascular disease, and 2.7% with renal dysfunction (serum creatinine >200 mmol and/or dialysis support). These differences in patient characteristics could help explain the differences between these studies.

The work by Brener and associates [16] implied that only patients with large cardiac enzyme release (>10 times ULR) had an association with increased mortality. No association between >5 times ULR and mortality was found, which is at odds with the studies by Klatte [14] and Costa [15], and differs significantly from our own findings.

Marso and colleagues [17] found similar results to our findings, demonstrating the importance of cardiac enzyme release >3 times ULR on increased mortality post-CABG. This association was still evident even after 5-years follow-up. The study recommended the routine data collection of CK-MB following CABG to aid in long-term risk assessment, which we would agree with.

The editorial by Mahaffey and Alpert [18] stressed the importance of developing a consensus about what constitutes a myocardial infarction post-CABG, especially since the joint European Society of Cardiology and American College of Cardiology Committee provided no standard criteria due to the lack of definitive data [12]. Our findings and recent other studies [14,15,17] provide such data, which will help lead toward such a consensus opinion.

The message from this study is clear: myocardial necrosis following CABG is undesirable. We have demonstrated that levels of CE release previously thought to be innocuous are independently associated with increased one-year mortality. CK-MB release three or more times above the upper limit of reference range, measured within the first 24 hours following isolated CABG should be regarded as prognostically significant and may form the basis for a simplified definition of peri-operative myocardial infarction following coronary artery bypass surgery.

## Authors' contributions

N Newall designed the study, outlined the analytical approach, and wrote the manuscript. AY Oo, RH Stables, BM Fabri, DR Ramsdale amended the study design and data interpretation. ND Palmer, T Hine collated, and validated biochemical data blind to clinical outcome and contributed to data interpretation. AD Grayson performed the statistical analysis and co-wrote the manuscript. All authors contributed to interpretation of data and reviewed the final report.

## Competing interests

The author(s) declare that they have no competing interests.

## Role of the funding source

Dr Newall was funded by the Merseybeat Appeal and has received project grants from Bristol Myers Squibb/Sanofi Synthelabo. The funding source had no role in the design, data collection, interpretation and writing of the manuscript.

## References

[B1] Califf RM, Abdelmeguid AE, Kuntz RE (1998). Myonecrosis after revascularisation procedures. J Am Coll Cardiol.

[B2] Ellis SG, Chew D, Chan A (2002). Death following creatine kinase-MB elevation after coronary intervention: importance of creatine kinase-MB level, completeness of revascularisation, ventricular function and probable benefit of statin therapy. Circulation.

[B3] Czerny M, Baumer H, Kilo J (2000). Inflammatory response and myocardial injury following coronary artery bypass grafting with or without cardiopulmonary bypass. European Journal of Cardio-thoracic surgery.

[B4] Topol EJ, Yadav SJ (2000). Recognition of the importance of embolisation in atherosclerotic vascular disease. Circulation.

[B5] Rasmussen C, Thiis JJ, Clemmensen P (1997). Significance and management of early graft failure after coronary artery bypass grafting. Feasibility and results of acute angiography and re-re-vascularisation. European Journal of Cardio-thoracic Surgery.

[B6] Lee ME, Sethna DH, Conklin CM (1983). CK-MB release following coronary artery bypass grafting in the absence of myocardial infarction. Ann Thorac Surg.

[B7] Svedjeholm R, Dahlin LG, Lundberg C (1998). Are electrocardiographic Q-wave criteria reliable for diagnosis of peri-operative myocardial infarction after coronary surgery?. Eur J Cardiothoracic Surg.

[B8] Brasch AV, Khan SS, Denton TA (2000). Twenty-year follow-up of patients with new peri-operative Q waves after coronary artery bypass grafting. Am J Cardiol.

[B9] Steuer J, Hörte LG, Lindahl B (2002). Impact of peri-operative myocardial injury on early and long-term outcome after coronary artery bypass grafting. Eur Heart J.

[B10] Chaitman BR, Alderman EL, Sheffield LT (1983). Use of Survival Analysis to Determine the Clinical Significance of New Q Waves After Coronary Bypass Surgery. Circulation.

[B11] Birdi I, Angelini GD, Bryan AJ (1997). Biochemical Markers of Myocardial Injury During Cardiac Operations. Ann Thoracic Surg.

[B12] The joint European Society of Cardiology/American College of Cardiology Committee (2000). Myocardial infarction redefined-A consensus document of the joint European Society of Cardiology/American College of Cardiology committee for the redefinition of myocardial infarction. J Am Coll Cardiol.

[B13] Newby LK, Alpert JS, Ohman EM (2002). Changing the diagnosis of acute myocardial infarction: Implications for clinical practice. Am Heart J.

[B14] Klatte K, Chaitman BR, Theroux P (2001). Increased mortality after coronary artery bypass graft surgery is associated with increased levels of postoperative creatine kinase – myocardial band isoenzyme release: results from the GUARDIAN trial. J Am Coll Cardiol.

[B15] Costa MA, Carere RG, Lichtenstein SV (2001). Incidence, predictors, and significance of abnormal cardiac enzyme rise in patients treated by bypass surgery in the arterial revascularization therapies study (ARTS). Circulation.

[B16] Brener SJ, Lytle BW, Schneider JP (2002). Association between CK-MB elevation after percutaneous or surgical revascularisation and three-year mortality. J Am Coll Cardiol.

[B17] Marso SP, Bliven BD, House JA (2003). Myonecrosis following isolated coronary artery bypass grafting is common and associated with an increased risk of long-term mortality. European Heart Journal.

[B18] Mahaffey KW, Alpert JS (2001). Cardiac enzyme elevation after cardiac surgery: the cardiologists perspective. Am Heart J.

[B19] French JK, White HD (2004). Clinical implications of the new definition of myocardial infarction. Heart.

[B20] Wynne-Jones K, Jackson M, Grotte G (2000). Limitations of the Parsonnet score for measuring risk stratified mortality in the north west of England. Heart.

[B21] Kaplan EL, Meier P (1958). Nonparametric estimation from incomplete observations. J Am Stat Assoc.

[B22] Cox DR (1972). Regression models and life-tables. J R Stat Soc.

[B23] Hosmer D, Lemeshow S (1989). Applied logistic regression.

[B24] O'connor GT, Morton JR, Diehl MJ (1993). Differences between men and women in hospital mortality associated with coronary artery bypass grafting. Circulation.

[B25] Januzzi JL, Lewandrowski K, MacGillivray TE (2002). A comparison of cardiac troponin T and creatine kinase-MB for patient evaluation after cardiac surgery. J Am Coll Cardiol.

[B26] Carrier M, Pellerin M, Perrault LP (2000). Troponin levels in patients with myocardial infarction after coronary artery bypass grafting. Ann Thorac Surg.

[B27] Selvanaygam JN, Pigott D, Balacumaraswami L (2005). Relationship of Irreversible Myocardial Injury to Troponin I and Creatine Kinase – MB Elevation After Coronary Artery Bypass Surgery: Insights From Cardiovascular Magnetic Resonance Imaging. JACC.

[B28] Holmvag L, Jurlander B, Rasmussen C (2002). Use of biochemical markers of infarction for diagnosing perioperative myocardial infarction and early graft occlusion after coronary artery bypass surgery. Chest.

[B29] Thielman M, Massoudy P, Marggraf G (2004). Role of troponin I, myoglobin, and creatine kinase for the detection of early graft failure following coronary artery bypass grafting. European Journal of Cardio-Thoracic Surgery.

